# Dr. Huidekoper Withdraws from the Veterinary Department of the University of Pennsylvania

**Published:** 1890-01

**Authors:** 


### DR. HUIDEKOPER WITHDRAWS FROM THE VETERINARY DEPARTMENT OF THE UNIVERSTY OF PENNSYLVANIA.

About ten years ago steps were taken to found a Veterinary Department at the University of Pennsylvania, two of the trustees being actively interested in the project. Rush Shippen Huidekoper, M.D., who was then assistant demonstrator of Surgery in the Medical Department, and an assistant surgeon in the dispensary service of the University Hospital, was offered the organization of the new department. He immediately went abroad at his own expense, and spent two years preparing himself for his future position. He obtained his degree as Veterinarian at Alfort, France, and then passed a year in practical work, and learning the methods of administration of the veterinary schools of Berlin, Vienna, Italy, and Lyons, besides visiting most of the other veterinary schools of Europe. In 1883, the first buildings of the veterinary department were completed, with funds furnished by the liberality of J. B. Lippincott, Esq., one of the trustees of the University, and Joseph E. Gillingham, Esq.

The department was organized, and Dr. Huidekoper was appointed Dean of the Veterinary Faculty, Professor of Internal Pathology, Professor of Zootechnics, and *pro tempore* Professor of Veterinary Anatomy. He was told that he would have a salary the first year of $1500, but the income of the department did not meet its expenses, the illness of Mr. Lippincott prevented him from taking the active interest he desired, and no funds were available for any salaries. The following year the death of Mr. Lippincott threw the department on its own resources. The faculty gave their services without compensation; however, all but three (Drs. Huidekoper, Zuill and Smith) received salaries from the other departments of the University, of whose faculties they were already members. With the second year, a Veterinary Hospital was needed, and as the Trustees of the University could not provide means for its erection, they authorized a loan, which was procured almost entirely by Dr. Huidekoper from his personal friends. This loan was charged against the receipts of the Veterinary Department, which has since then paid yearly 5 per cent. interest and, when able to, 5 per cent of the principal, both payments taking precedence of salaries.

The school gained yearly in number of students, viz: 1884-1885, 33, 1885–86 42, 1886–87 49, 1887–88 57, 1888–89 62 students, and the class of students improved in quality. In 1887 Dr. Huidekoper procured, mainly through his own efforts, an appropriation of $50,000 from the State Legislature, which was, however, vetoed by His Excellency, the Governor. In 1889, after still greater personal work, he again secured an appropriation of $50,000, to which was attached a proviso for twelve free scholarships, but the amount was reduced by veto of the Governor to $25,000.

In the autumn of 1887, Dr. Huidekoper made a full report to the Trustees of the University,[1] stating the needs and prospects of the school and asked an investigation of its condition with a view of remedying its defficiencies, but he was not aware that more than one of the trustees ever came near the institution, or asked for any information in regard to it. In January, 1889, Dr. Huide-

[1] Vide Annual Report of the Provost of the University of Pennsylvania for the year ending October 1, 1887.

koper tendered his resination from the positions he held, on the ground that his "means did not permit him to continue longer to devote the greatest part of his time[1] and his greatest energies to a position unattended by a salary. He was urged to delay his resignation until July, when it was accepted with the following letter :—

1811 SPRUCE STREET,
PHILADELPHIA.

RUSH SHIPPEN HUIDEKOPER, ESQ., M.D.

DEAR SIR :—Your resignation of your chair in the Veterinary Department of the University of Pennsylvania has now been before the Trustees since the beginning of the year. I induced you to allow action to be post. poned, hoping that a liberal appropriation by the State Legislature, and some liberal action on the part of the community, who should be widely and actively interested in the work of this department, would enable it to be placed upon such basis that the labors of yourself would be remunerated at least in some degree.

It is chiefly due to your enlightened views and self sacrificing energy that this school of Veterinary Medicine is already organized as the best in existence in any English speaking country. It certainly is not creditable to the City and State that a school which so admirably represents interests of such magnitude should, since the liberal gift of the late Mr. J. B. Lippincott and of Mr. Joseph E. Gillingham at the time it was started, have received such small and reluctant recognition from any except the family of its chief founder, Mr. Lippincott.

You and other members of the Veterinrry Faculty have labored for five years, and have yet received no salaries. The financial result of the last year makes it impossible to promise any better result in the near future. It is impossible to ask or expect you to continue to work as you have done without adequate remuneration. Finding, therefore, that there is no immediate prospect of sufficient funds being placed in their hands for this specific purpose, the Board of Trustees have authorized me to accept your resignation, and at the same time to express their deep appreciation of the learning, ability, and high professional spirit which have distiuguished your occupancy of your Professorship in the Veterinary Department of the University.

The service rendered by you in placing Veterinary Medicine upon a scientific basis for the first time in America will ever be held in honorable and grateful remembrance.

I beg to remain, yours faithfully,

(Signed) WILLIAM PEPPER,
*Provost.*

(Signed) JESSE Y. BURK, *Secretary.*

[1] His positions demanded eight lectures a week, clinical instruction daily, superintendence of dissecting room and practical work, general direction of school, hospital and correspondence.

In September, Dr. Huidekoper offered to resume his work, if relieved of his chair of Veterinary Anatomy, but, as a reorganization of the department had been decided upon by the Trustees, by which the school, or didactic part of the department, was entirely separated from the hospital and farriery, he was reappointed only to the chairs of Internal Pathology and Zootechnics, and the former demonstrator of Chemistry, John Marshall, M.D., Nat. Sc.D., was appointed assistant Professor of Chemistry and Dean of the Veterinary Faculty. The hospital and farriery were placed under the control of a board of managers consisting of five trustees of the University, five gentlemen of Philadelphia and two of the the clinical staff.

By this arrangement, the school portion, under the new dean, was to receive the tuition fees and other items of income, and with its small cost of running was to show a surplus at once and furnish several thousand dollars with which to pay salaries; while the hospital and farriery, which had been the cause of deficit of the institution as a unit, was to be stayed up by the new board of managers.

At the opening session (Oct. 1889), a chair of Biology was added to the curriculum, the study of botany was transferred from the first, to the already overcrowded second year studies, additional force of employés was at the disposal of the new dean, and extensive alterations and improvements in buildings were ordered by him, presumably under instructions from the Provost of the University, without consultation with Dr. Huidekoper. The cost of improvements was to be paid from the funds which had been obtained from the Legislature. Notwithstanding this, Dr. Huidekoper continued his work until late in November when on his return from a temporary absence, he found his laboratory, which he had occupied since the foundation of the school, greatly disarranged and valuable skeletons in course of preparation, and other personal material removed to other rooms, and, upon questioning, was informed by the dean that three days previously a faculty meeting had resolved to transfer the laboratory to another member of the faculty, when he notified the Provost that he would sever his connection with the University at the commencement of the Thanksgiving recess.

